# Three-dimensional stability during orthodontic retention: A comparative analysis of conventional, CAD/CAM-fabricated, and robotically bent fixed retainers versus removable appliances

**DOI:** 10.1007/s00784-026-06929-1

**Published:** 2026-05-23

**Authors:** Jeannine Köck, Franziska Lang, Christian Niederau, Marta Rizk, Hannah Al-Sakati, Norbert Lang, Michael Wolf, Isabel Knaup

**Affiliations:** Department of Orthodontics and Orofacial Orthopedics, RWTH Aachen University Hospital, Pauwelsstr. 30, Aachen, 52074 Germany

**Keywords:** Orthodontic retention, Fixed lingual retainer, CAD/CAM retainer, Tooth movement, Three-dimensional analysis, Wire syndrome

## Abstract

**Objectives:**

To evaluate three-dimensional anterior tooth position changes during orthodontic retention and to compare the effectiveness of three distinct fixed retainer fabrication designs including robotically bent retainers alongside with removable retainers.

**Materials and methods:**

This retrospective cohort study included 113 patients (226 dental arches; mean retention period 1.3 ± 0.6 years). Combined fixed lingual plus removable retention (group 1, *n* = 148 arches) comprised CAD/CAM Memotain, conventional multistranded stainless-steel (Twistflex), and robotically bent retainers; group 2 (*n* = 78 arches) received removable retention only. Digital model superimposition using a three-dimensional tooth-specific coordinate system quantified rotational and translational tooth movements, Little’s Irregularity Index (LII), transverse arch widths, and bonding failures.

**Results:**

Combined fixed and removable retention showed higher stability than removable-only retention (55% vs. 33% stable arches). Removable retention increased the odds of moderate-to-severe instability (maxilla OR 4.37, 95% CI 1.74–10.98; mandible OR 14.35, 95% CI 3.24–63.56), whereas fixed lingual retention was protective (maxilla OR 0.23, 95% CI 0.09–0.58; mandible OR 0.07, 95% CI 0.02–0.31). Instability mainly involved canine rotations and vertical translations. Robotically bent and CAD/CAM Memotain retainers showed the lowest movement magnitudes and variability, with minimal LII increases. Intercanine and intermolar widths remained stable across fixed retainers. Bonding failures (25%) strongly predicted severe instability.

**Conclusions:**

Three-dimensional tooth movements during retention mainly involve canine rotations and vertical translations. All fixed retainer designs (Twistflex, CAD/CAM-fabricated, and robotically bent) provided greater stability than removable-only retention.

**Clinical relevance:**

All fixed retainer designs require regular monitoring of bonding integrity; digitally fabricated and robotically bent retainers demonstrated marginally lower dimensional variability, which may be clinically relevant over longer observation periods.

**Supplementary Information:**

The online version contains supplementary material available at 10.1007/s00784-026-06929-1.

## Introduction

Long-term stability of orthodontic treatment outcomes has become a central concern in contemporary orthodontics, particularly in the context of rising patient expectations for durable, aesthetic results and increasingly individualized treatment concepts. Although relapse tendencies after active treatment are well documented, there is still no universally accepted, evidence-based standard for retention [1].

In routine clinical practice, bonded lingual retainers are widely used to maintain anterior alignment and are commonly considered a preferred option for long-term orthodontic retention [2, 3]. Recent network meta-analytic evidence indicates that digitally fabricated and laboratory-bent multistranded stainless-steel retainers are particularly effective in preserving anterior alignment and intercanine width, whereas intermolar width appears largely independent of retainer design [3]. With ongoing technological advances in retainer fabrication, an increasing variety of digitally planned and machine-assisted systems has been introduced into clinical practice, including workflows based on indirect bonding techniques [4]. However, despite their growing adoption, robust clinical data evaluating these newer fabrication approaches, particularly with respect to three-dimensional tooth movements during retention and retainer-related failure patterns, remain limited, underscoring the need for further longitudinal investigations.

At the same time, there is growing awareness that fixed retention may not always act as an entirely passive measure [5]. Bonded retainers are valued for their capacity to help stabilize tooth position, their inconspicuous appearance and their relative independence from patient compliance [6, 7]. Nevertheless, several reports have described clinically relevant, unintended tooth movements occurring in the presence of apparently intact retainers [5, 8–12]. So-called “active” retainers are capable of exerting forces on teeth and have been associated with characteristic patterns of unwanted movement, including torque changes of adjacent incisors (“X-effect”) and opposite inclinations of contralateral canines (“twist effect”) [8]. More recently, these observations have been summarized under the broader concept of “wire syndrome”, which encompasses X- and twist effects and other retainer-related changes [5]. It could be shown that wire syndrome represents a distinct form of retainer-associated misalignment that should be distinguished from physiological changes or classic relapse; in many of these cases, removal of the bonded retainer is required as an initial measure and, depending on the severity of the malalignment, subsequent orthodontic retreatment may be necessary [11, 13]. Furthermore, several studies indicate that retention behaviour differs between the maxilla and mandible [14, 15]. It could be shown that unwanted tooth movements and technical failures occur in the upper and in the lower arch, and that factors such as functional disturbances, oral habits and incomplete interincisal contacts may further increase the risk of anterior changes [14, 15]. In response, mixed retention strategies are commonly used in clinical practice, combining fixed and removable appliances within or between arches to enhance stability. Among removable retainers, Hawley plates and vacuum-formed retainers are most frequently prescribed and, despite differences in occlusal settling and comfort, both show acceptable short-term compliance [1, 16, 17].

The three retainer designs evaluated in this study differ systematically in wire geometry, material, and fabrication method. Conventional Twistflex retainers consist of triply stranded round stainless-steel wire (0.018 inch), whose helical architecture confers flexibility and allows physiological tooth mobility, but may also facilitate unintended force transmission under functional loading [18]. CAD/CAM-fabricated Memotain retainers are precision-cut from a NiTi sheet in a rectangular cross-Sect. (0.016 × 0.016 inch), exploiting superelastic material properties for passive lingual adaptation with high geometric reproducibility, at the cost of lower stiffness compared with stainless steel [10]. Robotically bent retainers, fabricated from spring-hard stainless-steel wire in either a square or round cross-section, combine computer-controlled bending precision with the mechanical robustness of conventional stainless steel; square cross-sections provide greater torsional resistance than round wires of equivalent diameter, which may contribute to improved rotational control of anterior teeth [19, 20].

While three-dimensional analyses of unwanted tooth movements have previously been conducted for conventional multistranded retainers [11–15], equivalent three-dimensional in vivo stability data are lacking for CAD/CAM-fabricated designs such as Memotain, whose clinical evaluation has focused on survival rates, bonding failures, and periodontal outcomes [10, 18, 21, 22], and are entirely absent for robotically bent fixed retainers, which have been assessed exclusively in vitro with respect to fabrication accuracy and interproximal fit [19, 20]. Robotically bent retainers occupy a distinct position within the retainer design spectrum: unlike Memotain, which achieves passive fit through the superelastic properties of a NiTi sheet, robotically bent systems apply computer-controlled precision bending to conventional stainless-steel wire, potentially combining the mechanical robustness of established wire materials with improved geometric reproducibility. The present study therefore includes robotically bent retainers as a third distinct design category to contribute to closing this evidence gap.

From a clinical perspective, retention outcomes vary substantially, and some patients develop moderate to severe tooth position changes during the retention phase. To better understand these clinically relevant cases, targeted analyses focusing on affected patients and their retention strategies are required. The aim of this retrospective study was to assess anterior tooth position changes in the maxilla and mandible during the short-/mid-term orthodontic retention phase following fixed appliance therapy, with particular emphasis on moderate and severe changes. Associations with the applied retention strategy and different fixed retainer designs, including CAD/CAM retainers, were evaluated. We hypothesized that anterior tooth position changes during orthodontic retention are influenced by the applied retention strategy and retainer design.

## Materials and methods

### Study design

This single-center retrospective cohort study was conducted in the Department of Orthodontics at the University Hospital of RWTH Aachen, Germany. The cohort comprised patients treated between 2018 and 2025 with follow-up in the retention phase. The protocol and use of routinely collected clinical data were approved by the Ethics Committee of the Medical Faculty of RWTH Aachen University (EK 25–302). Reporting adheres to the STROBE statement for cohort studies (Suppl. Table 1).

### Study population and eligibility criteria

**Fig. 1:**
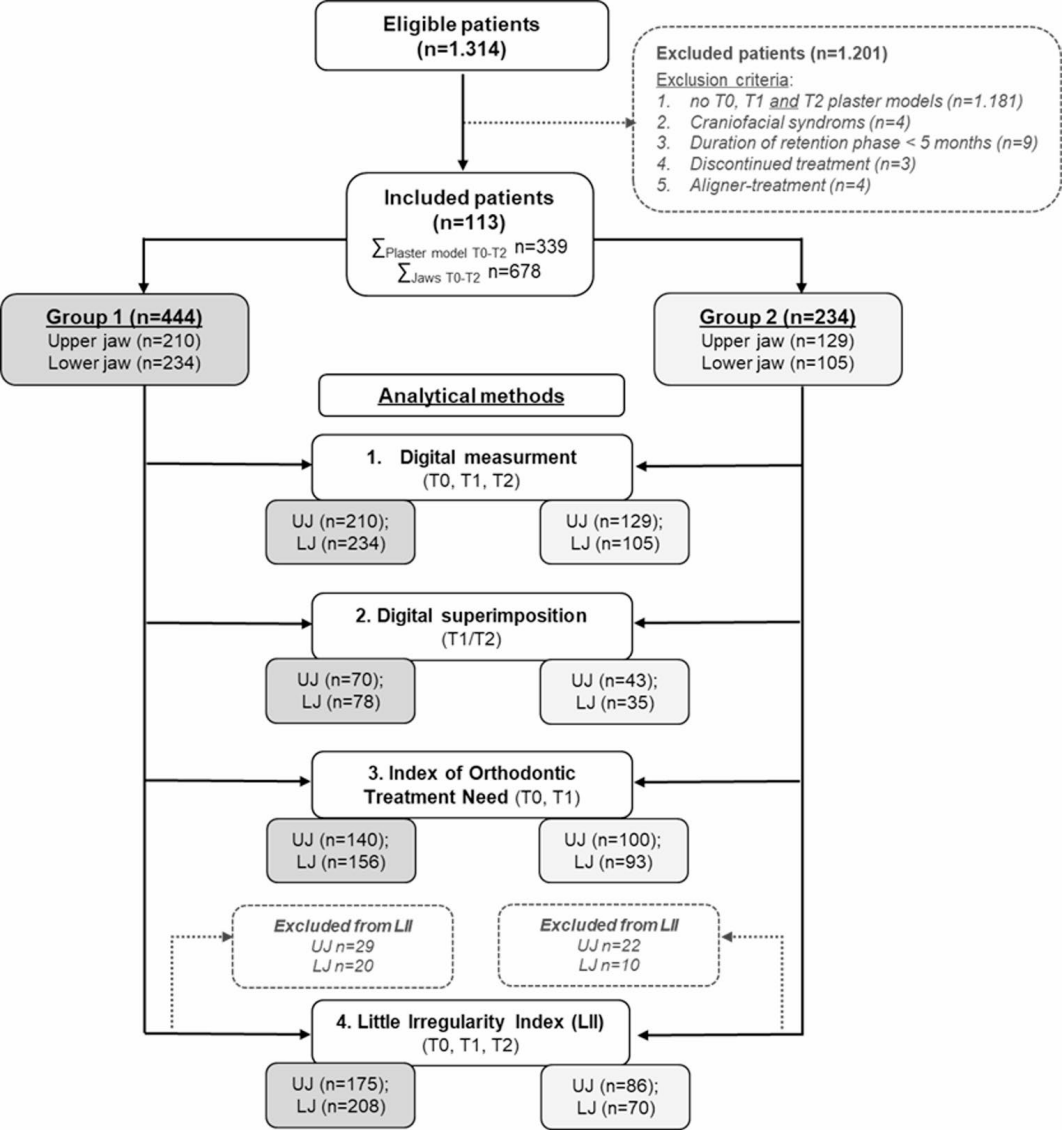
Flow diagram of the retrospective study cohort. Patients treated with both removable and fixed retainers were allocated to group (1) Patients who wore exclusively removable retainers due to refusal of a fixed retainer were allocated to group (2) UJ, upper jaw; LJ, lower jaw

Orthodontic diagnostic records of 1,314 consecutive patients treated at the department were screened for eligibility (Fig. 1). Inclusion criteria required the availability of plaster models at T0 (pre-treatment), T1 (debonding/completion of fixed appliance therapy), and T2 (retention), with a minimum interval of 5 months between T1 and T2. Eligible patients had been treated using either vestibular fixed appliances (slot 0.022 × 0.028 inch; *n* = 108) or a lingual appliance system (slot 0.018 × 0.026 inch; *n* = 5). Exclusion criteria included missing study models at any time point, craniofacial syndromes, a retention period of less than 5 months, discontinued treatment, or aligner therapy.

### Orthodontic retention protocols

As part of standard clinical care, all patients received removable thermoplastic retainers in both arches immediately at debonding to maintain tooth position during the interim period until delivery of a Hawley retainer. Hawley retainers were provided four weeks later as the standard long-term removable retention option and were likewise prescribed bilaterally. When clinically indicated, functional appliances were prescribed instead of Hawley retainers. Patients were instructed to wear the removable retainers full-time during the initial 6 months (including night-time and daytime when at home), followed by a reduction to night-time wear only, with a further gradual reduction in frequency thereafter.

When fixed lingual retention was applied, the following retainer systems were used: conventional multistranded stainless-steel Twistflex retainers (Advanced Orthodontics Näpflein GmbH, Düsseldorf, Germany; 0.018-inch, triply stranded) and two-dimensional digitally fabricated retainers, including CAD/CAM-fabricated Memotain retainers (Scheu-Dental, Iserlohn, Germany; 0.016 × 0.016-inch nickel–titanium) and two-dimensional robotically bent retainers produced using the Bender system (ortho Penthin, Schwanewede, Germany; remanium stainless steel wires by Dentaurum, Ispringen, Germany; square 0.016 × 0.016-inch or round 0.016-inch, spring-hard). CAD/CAM-fabricated Memotain retainers were placed using an indirect bonding technique [10] (Table 1).

**Table 1:** Types of fixed retainers applied in group 1

Retainer type	Material	Structure design	Diameter	*n*	Manufacturer
Twistflex	Stainless steel	Triple-stranded twisted	0.018“	50	Advanced Orthodontics Näpflein GmbH, Düsseldorf, Germany
Multiflex	Stainless steel	Eight-strand twisted	0.017“ x 0.025“	18	GC Orthodontics Europe GmbH. Breckerfeld, Germany
Memotain	Nickel-titanium alloy	Square wire	0.016“ x 0.016“	42	Scheu-Dental GmbH. Iserlohn, Germany
Robotic bent retainer	Stainless steel	Round or square wire	0.016“/ 0.016“ x 0.016“	38	Bender Robot, Ortho Pentin GmbH, Germany Dentaurum GmbH & Co. KG. Ispringen, Germany

Dental arches were classified post hoc into two retention exposure groups. Group 1 consisted of patients with combined retention (any type of fixed lingual retainer including conventional Twistflex, CAD/CAM-fabricated Memotain, or robotically bent designs plus removable retainer). Group 2 consisted of patients with removable retention only (fixed lingual retainer was declined by the patient and/or the caregivers). Because group 1 pools all fixed retainer designs under a single exposure category, the primary fixed-versus-removable comparison reflects a mixed fixed-retainer protocol; comparisons among individual retainer designs are reported as secondary subgroup analyses.

### Data acquisition and model analysis

**Fig. 2:**
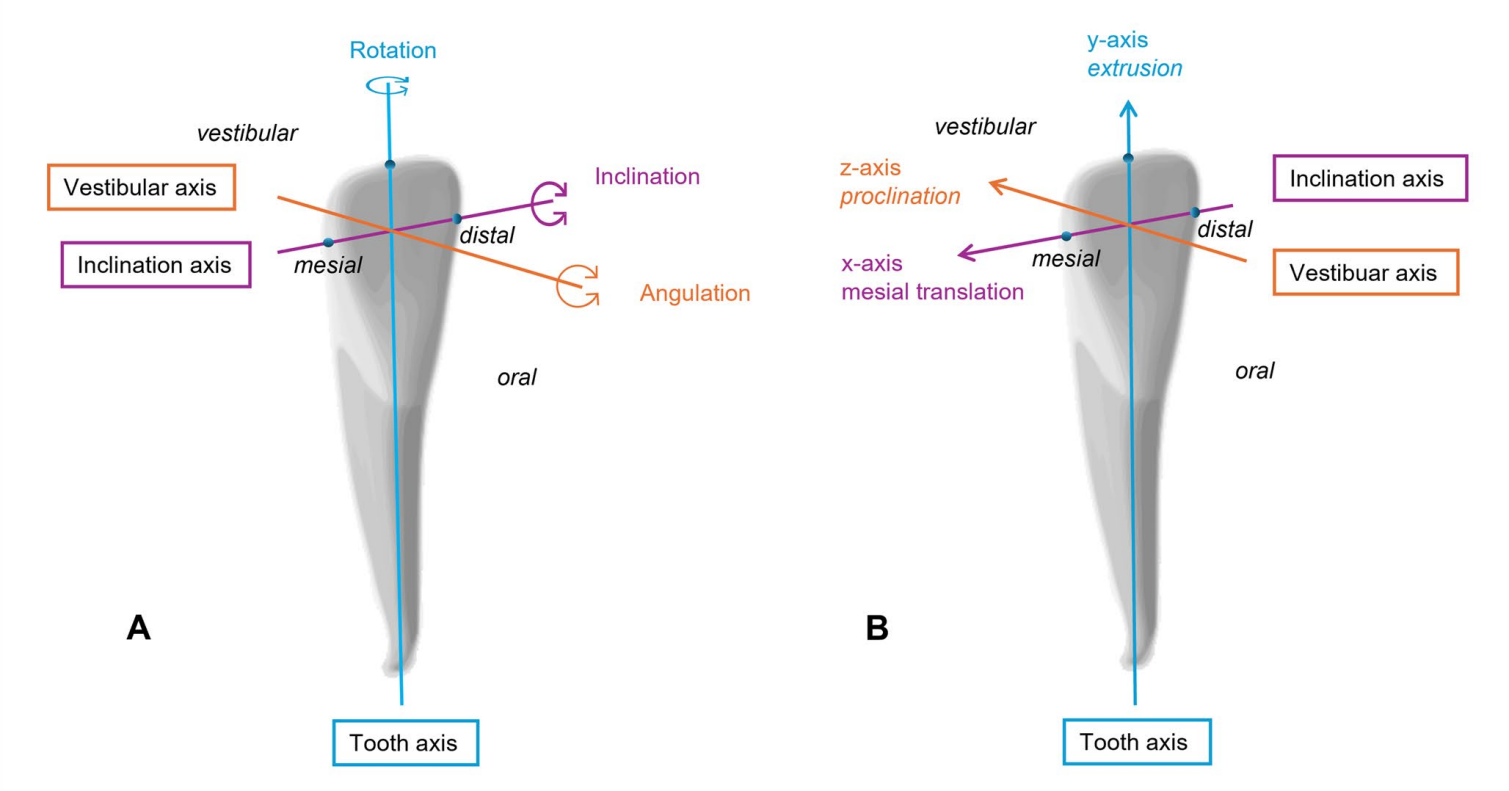
Schematic illustration of the tooth-specific local anatomical coordinate system used for assessment of (**A**) rotational and (**B**) translational tooth movements in the OnyxCeph software. The coordinate system is based on the definitions provided in OnyxWiki

Paper charts and electronic health records were systematically reviewed to extract patient characteristics and retention-related exposures relevant to the study objectives. Retention-related variables after T1 included the retention protocol (fixed lingual retainer combined with removable retainers at the tooth level using the 3D software OnyxCeph3™ (Image Instruments, Chemnitz, Germany). Digital models from T1 and T2 were superimposed using a tooth-specific local anatomical coordinate system based on three orthogonal axes: the x-axis (inclination/arch axis) connecting the mesial and distal contact points, the y-axis (tooth/rotation axis) extending from the incisal point through the midpoint of the mesiodistal line, and the z-axis (vestibular/angulation axis) oriented perpendicular to the x-y plane (Fig. 2).

Rotational movements were recorded in degrees (°) around each axis inclination (x; positive = buccal/vestibular tipping), rotation (y; positive = mesial rotation of the labial surface), and angulation (z; positive = mesial incisal and distal apical displacement), while translational movements were recorded in millimeters (mm) along the same axes translation (x; positive = mesial, negative = distal), translation (y; positive = extrusion, negative = intrusion), and translation (z; positive = proclination, negative = retroinclination).

**Fig. 3:**
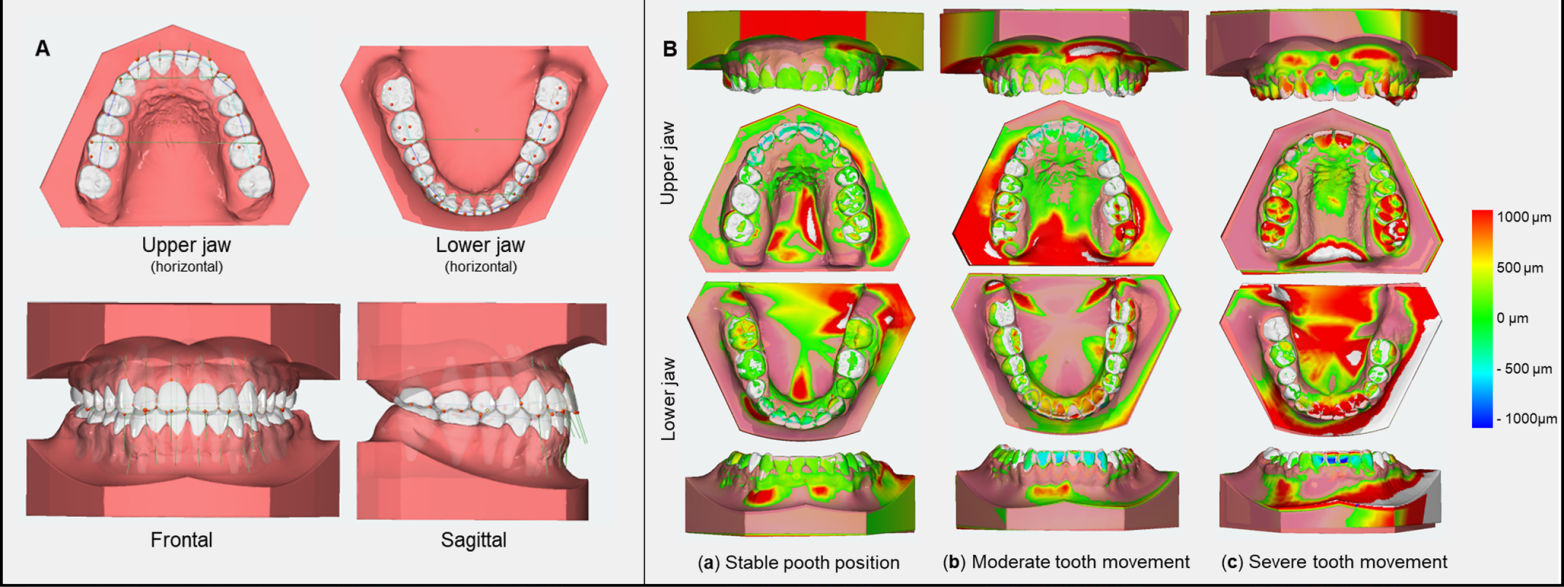
Digital model measurement and superimposition for assessment of tooth movement during the orthodontic retention phase. (**A**) Representative example of tooth-level digital model measurement after removal of multibracket appliances (T1). (**B**) Representative superimposition of digital models from T1 and T2 of the upper and lower dental arches from three different patients illustrating (**a**) stable tooth positions, (**b**) moderate tooth movement, and (**c**) severe tooth movement during retention. Tooth movements were assessed using the Aligner module of the 3D software OnyxCeph3™ (Image Instruments, Chemnitz, Germany), based on a tooth-specific local anatomical coordinate system. The color gradient from blue to red indicates increasing magnitude of tooth movement (+ 1000 μm to −1000 μm)

Superimposition was performed using an Iterative Closest Point (ICP) best-fit algorithm (Fig. 3), constrained by stable reference structures (mandibular first molars and premolars; palatal rugae and median raphe in the maxilla) based on previous works [13, 23, 24]. Each arch was superimposed ten times to calculate mean rotational and translational tooth movements, including only erupted teeth. Method error was assessed in twelve randomly selected patients and showed low SEM values (0.1° for rotations, 0.02 mm for translations), indicating high measurement accuracy.

The primary outcome was the severity of anterior tooth movements during retention (T1–T2), defined as an arch-level ordinal variable based on the maximum tooth-level movement. Severity was categorized as stable (< 5° and ≤ 0.5 mm), moderate (5°–9° and > 0.5–≤ 1.0 mm), or severe (> 9° and > 1.0 mm), with classification based on the higher severity of either rotation or translation as applied in prior studies [13, 25, 26].

### Analysis of transverse dental dimensions and anterior alignment

All orthodontic study models obtained at T0–T2 were analysed to assess changes in tooth position during the retention period and to evaluate the effects of different retention protocols on post-treatment stability. Model analysis was performed using the semi-automated landmark-based analysis module of OnyxCeph^3^™ (Image Instruments, Chemnitz, Germany; Module Analysis, patch “Zahnbreitenanalyse Aachen”), with automatically suggested landmarks verified and, if necessary, manually corrected based on previous works (Fig. 3) [27]. Linear distances were recorded in millimeters (mm), either parallel or perpendicular to the occlusal plane. All models were measured ten times on different days by a trained examiner (JK), yielding excellent intrarater reliability (ICC = 1.00). Analyses focused on models obtained after completion of active orthodontic treatment (T1) and during the retention phase (T2), while T0 measurements were used for baseline description only; the measured parameters are detailed below.

**Fig. 4:**
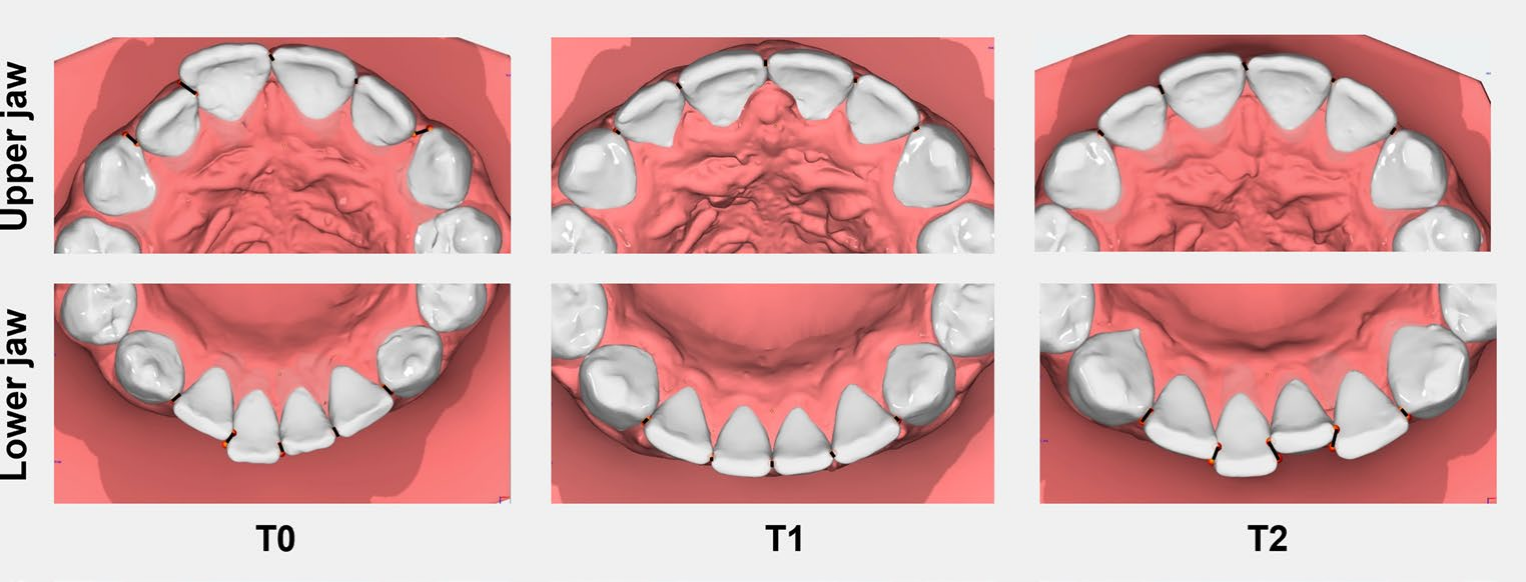
Assessment of Little’s Irregularity Index. Representative measurements of Little’s Irregularity Index in the upper dental arch (top row) and lower dental arch (bottom row) of a patient at the start of treatment (T0), after removal of the multibracket appliance (T1), and during the retention phase (T2)

- Intercanine width was defined as the linear distance between the cusp tips of the canines. The reference point was placed centrally at the highest point of the canine cusp. In cases of cusp tip wear, the midpoint of the worn surface was used. If the canine was unerupted or positioned ectopically, the reference point was positioned centrally on the alveolar ridge based on previous works [28–31].
- Intermolar width was defined as the distance between the mesiobuccal cusp tips of the first molars in the maxilla and mandible. In cases of cusp wear, the midpoint of the mesiobuccal cusp was used as the reference point. If the first molar was missing and space closure had occurred by mesialization of the second molar, the intermolar width was measured at the mesiobuccal cusp tip of the second molar based on previous works [28, 32].
- Little’s Irregularity Index (LII) was used to quantify anterior tooth irregularity in the maxilla and mandible by summing the horizontal displacements between anatomic contact points, measured parallel to the occlusal plane [33]. Measurements were performed at T0, T1, and T2; jaws with incompletely erupted or congenitally missing anterior teeth at T0 were excluded (Fig. 4). Intrarater reliability showed excellent agreement (ICC = 0.99).

### Statistical analysis

Data preparation was performed in Microsoft Excel 365 (Microsoft Corp., Redmond, WA, USA). Descriptive statistics, statistical analyses and figures were generated using GraphPad Prism (v10.6.1; GraphPad Software, San Diego, CA, USA). Intraclass Correlation Coefficient was conducted in IBM SPSS Statistics (v31.0; IBM Corp., Armonk, NY, USA). Normality of continuous variables was assessed using the Shapiro-Wilk test. Depending on the distribution, appropriate parametric (one-way ANOVA) or non-parametric tests (Mann-Whitney U test, Kruskal-Wallis test) were applied. Categorical variables were analyzed using the chi-square test. Significant Kruskal-Wallis results were followed by Dunn’s post-hoc test. Statistical significance was set at a two-sided α = 0.05.

The primary outcome was the severity of anterior tooth position changes during the retention phase, categorized as stable, moderate, or severe and defined by the maximum rotational or translational movement of the anterior teeth per arch from T1 to T2. Associations between the primary outcome and prespecified explanatory variables were analyzed using proportional-odds ordinal logistic regression. Covariates were selected a priori based on clinical relevance and refined using backward elimination. Effect estimates are reported as odds ratios with 95% confidence intervals, and model assumptions and fit were evaluated using standard diagnostic procedures. Secondary outcomes included changes in intercanine and intermolar width and Little’s Irregularity Index, as well as associations with demographic variables (age, sex) and treatment-related factors (treatment duration, retention protocol, and bonding failures of fixed retainers). These covariates were included to adjust for confounding.

## Results

### Study cohort and observation period

A total of 113 patients (65 females, 48 males) fulfilled the inclusion criteria and contributed 226 dental arches for analysis. Mean age at treatment start was 16.5 ± 6.6 years, mean duration of active orthodontic treatment 33.2 ± 14.0 months, and mean retention period 1.3 ± 0.6 years. According to the retention protocol, 148 arches were assigned to fixed lingual plus removable retention (group 1) and 78 arches to removable-only retention (group 2). Maxillary and mandibular arches were analyzed separately (Fig. 1).

### Severity of tooth position changes during retention

**Fig. 5:**
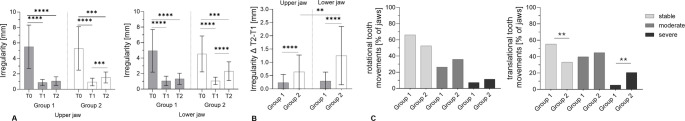
(**A**) Comparison of Little’s Irregularity Index (LII) between group 1 (upper jaw *n* = 67; lower jaw = 75) and group 2 (upper jaw *n* = 40; lower jaw *n* = 35) at the start of treatment (T0), after removal of the multibracket appliance (T1), and at the end of the retention phase (T2). (**B**) Significant increase of LII between T1 and T2 in group 2. Mean ± SD; **p < 0.01, ***p < 0.005, ****p < 0.0001 (Kruskal-Wallis test, Mann-Whitney U test). (**C**) Quantification of tooth alignment in group 1 versus group 2 stratified by rotational and translational tooth movements during the retention phase (T1/T2). For each group, the proportions of jaws with stable outcomes, moderate malocclusion, and severe malocclusion were normalized to 100%; **p < 0.01 (chi-square test with Bonferroni correction)

Overall, most arches remained stable or showed only moderate tooth position changes during retention (Table 2). In group 1, 55% of arches were classified as stable, 38% as moderate, and 7% as severe with regard to translational movements. In group 2, stability was observed in 33%, moderate changes in 45%, and severe changes in 21% of arches. Severe changes were more frequent in the mandible than in the maxilla (19% vs. 15%). Differences between group 1 (fixed lingual plus removable retention) and group 2 (removable-only retention) were more pronounced for translational movements. Here, the distribution of tooth alignment differed significantly between the groups (χ²(2) = 16.58, *p* = 0.0003). Post-hoc pairwise comparisons with Bonferroni correction revealed that Group 1 showed a higher proportion of stable alignment, whereas Group 2 exhibited a higher proportion of severe misalignment (both *p* < 0.01). No significant difference was observed for moderate misalignment (Fig. 5C).

**Table 2:** Demographic characteristics of dental arches according to tooth stability during orthodontic retention

Dental arches, *n* (%)	Total	Stable (≤ 5°/< 0.5 mm)	Moderate (≥ 5- ≤ 9°/ > 0.5 mm ≤ 1.0 mm)	Severe (> 9°/> 1.0 mm)	*p*-value
	226 (100)	87 (38.5)	101 (44.7)	38 (16.8)	
Female, *n (%)*	130 (100)	51 (39.2)	56 (43.1)	23 (17.7)	0.819^§^
Male, *n (%)*	96 (100)	36 (37.5)	45 (46.9)	15 (15.6)	
Age at T0 *(years; mean ± SD)*	15.2 ± 6.8	16.2 ± 7.7	14.7 ± 6.4	14.1 ± 5.5	0.293^$^
Age at T1 *(years; mean ± SD)*	19.3 ± 6.5	20.2 ± 7.3	19.1 ± 6.1	18.0 ± 5.6	0.133^$^
Age at multibracket insertion *(years; mean ± SD)*	16.5 ± 6.6	17.5 ± 7.4	16.0 ± 6.2	15.4 ± 5.4	0.142^$^
Duration multibracket treatment *(months; mean ± SD)*	33.3 ± 13.9	32.1 ± 10.9	35.6 ± 15.6	29.9 ± 14.2	0.116^$^
Duration retention phase *(years; mean ± SD)*	1.3 ± 0.6;	1.1 ± 0.4	1.3 ± 0.5	1.7 ± 0.8****	< 0.0001^$^

^§^Chi^2^-Test, ^$^Kruskal-Wallis-Test, ****p < 0.0001

### Rotational tooth movements (moderate and severe cases)

For the analysis of clinically relevant rotational deviations, only arches classified as moderate or severe were included and compared between group 1 (fixed + removable retention, *n* = 50) and group 2 (removable-only retention, *n* = 37). Movements were evaluated separately for maxilla and mandible and stratified by tooth type (canines, lateral incisors, central incisors).

**Fig. 6:**
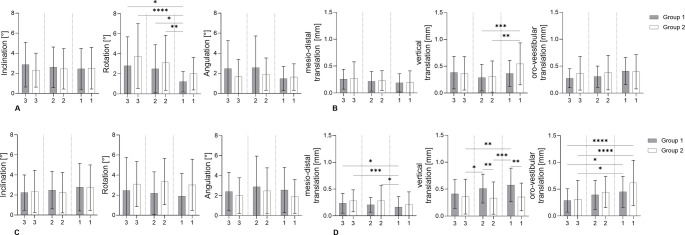
Comparison of moderate and severe rotational and translational tooth movements different retention protocols. (**A**/**C**) Rotational movements of central incisors (1), lateral incisors (2), and canines (3) along the x- (inclination), y- (rotation), and z-axis (angulation) in group 1 (fixed and removable retainers; *n* = 50) and group 2 (removable retainers; *n* = 37) in the upper (**A**) and lower jaw (**C**). (**B**/**D**) Translational movements along the x- (mesial/distal), y- (intrusion/extrusion), and z-axis (protrusion/retrusion) in group 1 (*n* = 67) and group 2 (*n* = 51) in the upper (**B**) and lower jaw (**D**). Data are presented as mean ± SD. *p ≤ 0.05, **p < 0.01, ***p < 0.005, ****p < 0.0001 (Kruskal-Wallis test)

In the maxilla (Fig. 6A) rotations around the y-axis (tooth rotation) showed the largest differences between retention protocols. Group 2 exhibited significantly higher rotational values than group 1 (*p* = 0,003), particularly for canines (group 1: 2.8 ± 2.9° vs. group 2: 3.8 ± 3.2°) and lateral incisors (2.5 ± 2.4° vs. 3.1 ± 2.7°). Rotations around the x-axis (inclination) were comparable between groups (canines: 2.6 ± 2.1° vs. 2.4 ± 1.9°), while z-axis (angulation) movements remained low overall, with slightly higher values in group 1 (mean 2.2°).

In the mandible **(**Fig. 6C**)**, rotational movements were predominantly observed around the y-axis (tooth rotation), whereas rotations around the x- (inclination) and z-axes (angulation) remained small in both groups. Group 2 tended to show higher y-axis rotations than group 1, particularly in the canines, where mean values were increased (group 1: 2.5 ± 3.3° vs. group 2: 3.1 ± 2.3°).

### Translational tooth movements (moderate and severe cases)

Translational movements were analysed using the same moderate-to-severe subset. Across both jaws, translations along the x-axis (mesial–distal) were the smallest, whereas y-axis (intrusion/extrusion) and z-axis (pro-/retrusion) movements were more pronounced.

In the maxilla (Fig. 6B) in- and extrusions were significantly greater in group 2, particularly for central incisors (group 1: 0.37 ± 0.24 mm vs. group 2: 0.54 ± 0.39 mm, *p* = 0.01). Similar trends were observed along the z-axis, with group 2 showing higher values across all tooth types (mean increase 0.38 mm). Mean translational movements along the x-axis remained low in both groups (< 0.25 mm) without significant differences.

In the mandible (Fig. 6D)., in- and extrusions were greater in group 1, particularly for the central incisors (group 1: 0.58 ± 0.31 mm vs. group 2: 0.35 ± 0.28 mm). Along the z-axis, translational movements (pro-/retrusion) increased from canines to central incisors in both groups, with values of 0.29 ± 0.22 mm versus 0.31 ± 0.35 mm for canines as well as 0.45 ± 0.29 mm versus 0.62 ± 0.42 mm for central incisors (group 1 vs. group 2). Mean translational movements along the x-axis remained low in both groups (< 0.23 mm) without relevant group differences.

### Predictors of instability

Ordinal logistic regression identified independent predictors of increased instability during retention (Table 3). Younger age at treatment start was associated with higher instability in the mandible (OR 0.93 per year; 95% CI 0.87–0.99). Longer retention intervals significantly increased the odds of moderate-to-severe changes (maxilla OR 4.06, 95% CI 1.89–8.74; mandible OR 2.72, 95% CI 1.30–5.72). Retention protocol had a strong effect: removable-only retention increased the odds of instability (maxilla OR 4.37, 95% CI 1.74–10.98; mandible OR 14.35, 95% CI 3.24–63.56), whereas fixed lingual retention reduced the odds of moderate-to-severe tooth position changes (maxilla OR 0.23, 95% CI 0.09–0.58; mandible OR 0.07, 95% CI 0.02–0.31).

**Table 3:** Ordinal logistic regression analysis of factors associated with the severity of tooth movement during the retention phase

Independent variables	Upper jaw				Lower jaw			
	Estimator (β-coefficient)	Significance (*p*-value of Wald test)	Odds ratio (e^β^)	Confidence interval of OR (lower; upper limit)	Estimator (β-coefficient)	Significance (*p*-value of Wald test)	Odds ratio (e^β^)	Confidence interval of OR (lower; upper limit)
Duration of retention phase (T2-T1) [years]	1.402	< 0.001	4.06	1.89–8.74	1.002	0.008	2.72	1.30–5.72
Age at the time of insertion of the multibracket appliance [years]	excluded from the final model^1^				−0.073	0.031	0.93	0.87–0.99
Removable retainer^2^	1.475	0.002	4.37	1.74–10.98	2.664	< 0.001	14.35	3.24–63.56
Fixed retainer^2^	−1.475	0.002	0.23	0.09–0.58	−2.664	< 0.001	0.07	0.02–0.31

^1^: Variable was excluded from the final model due to lack of significance

^2^: An ordinal logistic regression model (proportional odds model) was used. Retainer types were coded using the respective other retainer as reference category

### Little’s Irregularity Index

Baseline Little’s Irregularity Index (LII) did not differ between groups (Fig. 5). After treatment, alignment was largely maintained during retention. Mean LII changes from T1 to T2 were 0.4 ± 0.5 mm in the maxilla and 0.6 ± 0.8 mm in the mandible. Group 1 showed significantly smaller increases (maxilla 0.2 ± 0.3 mm, mandible 0.3 ± 0.3 mm) than group 2 (maxilla 0.6 ± 0.6 mm, mandible 1.3 ± 1.1 mm; *p* = 0.003). No significant differences in LII changes were observed between fixed retainer types. This suggests that the observed superiority in stability is associated with the use of fixed retention itself rather than the specific retainer design.

### Retainer type and three-dimensional tooth movements

However, as Little’s Irregularity Index may not capture subtle differences, additional analyses of rotational and translational tooth movements were performed to compare fixed retainer designs. All dental arches across the full severity spectrum (stable to severe tooth position changes) were included and the removable retention group (red line) was added as a reference for comparison. Mean rotational movements ranged between 1.2° and 2.1° on the tooth level, while translational movements ranged between 0.13 and 0.39 mm, depending on axis and jaw.

**Fig. 7:**
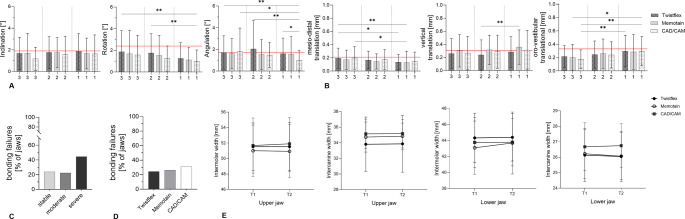
(A/B) Quantification of rotational and translational tooth movements according to retainer type. (**A**) Rotational movements of central incisors (1), lateral incisors (2), and canines (3) along the x- (inclination), y- (rotation), and z-axis (angulation) in patients treated with Twistflex (*n* = 68), Memotain (*n* = 38), and robotic bent retainers (*n* = 42). (**B**) Translational movements along the x- (mesial/distal), y- (intrusion/extrusion), and z-axis (protrusion/retrusion) in patients with Twistflex (*n* = 68), Memotain (*n* = 38), and robotic bent retainers (*n* = 42) with the red line indicating the mean values of the removable-only retention group (reference group, *n* = 78). Data are presented as mean ± SD. *p ≤ 0.05, **p < 0.01 (Kruskal-Wallis test). (**C**) Percentage of patients exhibiting retainer bonding failures according to tooth movement severity during the retention phase, categorized as stable tooth position, moderate, or severe tooth movement; chi-square test (*p* = 0.147; chi-square test). (**D**) Percentage of patients exhibiting retainer bonding failures according to Twistflex, Memotain and CAD/CAM retainer (*p* = 0.743; chi-square test. (**E**) Stabilization of intercanine and intermolar distances according to fixed retainer type. Data are presented as mean ± standard deviation. Statistical comparisons were performed using the Kruskal-Wallis test or one-way ANOVA

Compared with removable retention (red line), fixed retainers generally showed smaller rotational movements, indicating improved stabilization (Fig. 7A). Among fixed retainers, Memotain and robotically bent retainers exhibited comparable or slightly lower rotational movements than Twistflex retainers, reaching statistical significance only for the angulation of the central incisors (Fig. 7A). Across translational movements no statistically significant differences could be observed **(**Fig. 7B**).**

Although no fixed retainer completely prevented three-dimensional tooth movement, digitally fabricated and robotically bent retainers tended to show the lowest variability, while all retainers remained below the values observed in the retention group with only removable retainer (reference group).

### Bonding failures and severity of relapse

Bonding failures occurred in 25% of arches with fixed retainers after a mean period of 14.7 ± 6.3 months. The majority of bonding failures (49%) occurred within the first six months of the retention phase, while 24% occurred within the first 12 months. A further 27% of bonded sites debonded after more than one year. Bonding failures were more frequent in arches with severe tooth position changes (44.4%) than in those with moderate (22.2%) or stable outcomes (23.8%) (Fig. 7C). Among cases with bonding failure, 24.2% occurred in Twistflex retainers, 26.3% in CAD/CAM-fabricated Memotain retainers, and 30.9% in robotically bent retainers **(**Fig. 7D).

### Intercanine and intermolar width stability

Intercanine and intermolar widths were largely preserved across all fixed retainer types in both jaws (Fig. 7E). In the maxilla, intermolar width at T1 ranged from 51.0 ± 3.3 mm to 51.7 ± 3.6 mm, with mean changes during the retention phase remaining below 0.3 mm for all fixed retainer designs. Maxillary intercanine width ranged from 33.8 ± 3.5 mm to 35.1 ± 1.9 mm and showed minimal changes at T2; CAD/CAM-fabricated Memotain retainers exhibited the lowest variability (mean Δ = 0.04 mm).

In the mandible, intermolar width remained highly stable (43.9 ± 3.2 mm at T1), with mean changes of less than 0.2 mm. Mandibular intercanine width demonstrated slightly greater variability but remained comparable across retainer types, again with marginally lower variability observed for CAD/CAM-fabricated and robotically bent retainers.

## Discussion

The present retrospective investigation evaluated three-dimensional tooth position changes during the orthodontic retention phase using digital model superimposition, with a particular focus on patients exhibiting moderate to severe post-treatment misalignment. Unlike many previous studies, the present analysis specifically targeted moderate to severe tooth movements during retention and examined the influence of different retention protocols and fixed retainer designs.

Although most patients maintained stable or only moderately altered tooth positions, clinically relevant tooth movements were observed during retention despite the presence of fixed lingual retainers. This finding is in line with previous clinical observations and systematic reviews indicating that bonded retainers do not guarantee absolute tooth immobilization and that unwanted tooth movements may occur in a small but clinically relevant proportion of patients [5, 8, 9, 34].

Reported prevalence rates of wire syndrome vary widely in the literature, ranging from approximately 1–15% when only clinically manifest X-effects are considered [9, 35, 36]. In contrast, 3D-based analyses demonstrate that subtle rotational and translational tooth movements occur far more frequently and may represent early stages of the same pathomechanism [13–15, 25]. These findings support the concept that clinically evident X-effects constitute a late manifestation of a continuum that begins with initially asymptomatic, retainer-associated tooth movements and may evolve over several years.

A key finding of this study is the strong association between the applied retention protocol and post-treatment stability. Removable-only retention was associated with substantially increased odds of moderate-to-severe tooth position changes, whereas fixed lingual retention was associated with significantly lower instability in both jaws, particularly in the mandible. It should be noted, however, that this comparison reflects a heterogeneous mixed fixed-retainer protocol in group 1, and that the observational design limits causal interpretation (see Limitations). A recent meta-analysis also shows that fixed retainers can offer advantages in terms of the stability of treatment results in complex cases such as extraction cases [37]. In addition, longer retention intervals were independently associated with increased instability, indicating that tooth movements may develop progressively over time and are not restricted to the early post-treatment phase. Similar long-term observations have been reported in studies on permanent retention and extended follow-up periods [38, 39].

Post-treatment changes were predominantly characterized by rotational instability and vertical or sagittal positional variability rather than by pronounced mesial-distal displacement. Rotational movements were most evident in the canine region, particularly in the maxilla, where patients treated with removable retention only showed greater loss of rotational alignment compared with those receiving combined fixed and removable retention. With regard to translational movements, mesial–distal displacements were minimal in both jaws, whereas vertical (intrusion/extrusion) and sagittal positional variability were more pronounced. Increased translational values along the vertical axis should therefore be interpreted as greater positional variability rather than a uniform directional tendency, as both intrusion and extrusion contributed to the measured values. These findings are in accordance with recent three-dimensional analyses demonstrating that rotational control of anterior teeth, especially canines, is more reliably maintained with bonded retainers and highlight the importance of vertical control during retention, particularly in patients with pre-existing vertical discrepancies [13–15].

Fixed retainers demonstrated superior stabilisation compared to removable retention. Therefore, a subsequent analysis was performed to analyse whether differences exist between specific fixed retainer designs. These retainers (conventional Twistflex, CAD/CAM-fabricated Memotain, and robotically bent retainers) differ with regard to material, wire geometry, and manufacturing methods, which may influence mechanical properties. These aspects have been discussed in the literature with respect to fabrication accuracy, passive adaptation, and potential clinical complications (Charavet et al. 2024, Knaup et al. 2021, Wolf et al. 2016, Wolf et al. 2015, Koller, Craveiro et al. 2023). However, differences in 3D stability between the evaluated designs were overall small and frequently non-significant in the present study; accordingly, no definitive conclusions regarding the superiority of any particular material or design can be drawn.

To our knowledge, the present study is the first to evaluate robotically bent fixed retainers within a three-dimensional in vivo tooth stability framework, alongside CAD/CAM-fabricated and conventional multistranded designs. Existing three-dimensional clinical analyses have been confined to conventional multistranded retainers [11–15]. For CAD/CAM-fabricated designs such as Memotain, clinical evidence addresses survival rates and periodontal outcomes [10, 18, 21, 22], but three-dimensional tooth movement data are absent; and for robotically bent retainers, published evidence remains limited to in vitro assessments of interproximal accuracy and bending reproducibility [19, 20]. The present findings demonstrate that robotically bent retainers achieve three-dimensional stability broadly comparable to Memotain, with both designs showing marginally lower movement magnitudes and dimensional variability than conventional Twistflex retainers, constituting the first in vivo evidence for their use as a precision alternative that combines the mechanical robustness of stainless-steel wire with the geometric reproducibility of digitally assisted fabrication.

Comparison of fixed retainer designs revealed that all analysed retainers were effective in maintaining intercanine and intermolar widths throughout the mid‑term retention period. Differences among retainer types were modest; however, two‑dimensional CAD/CAM‑fabricated Memotain retainers and robotically bent retainers exhibited slightly lower mean dimensional changes, reduced variability, and smaller increases in anterior irregularity compared with conventional multistranded Twistflex retainers.

These outcomes are in line with recent in vitro investigations demonstrating superior bending accuracy and reproducibility of digitally fabricated and robotically bent retainers relative to manually contoured multistranded wires [20, 40]. Clinically, overall post‑treatment stability and periodontal health appear to be largely comparable across different fixed retainer types, though CAD/CAM‑based systems have been associated with marginal gains in fit precision and mechanical consistency [41–43]. Recent systematic reviews and network meta‑analyses have likewise reported small yet consistent advantages of CAD/CAM retainers in maintaining anterior alignment and intercanine width, while intermolar width seems widely independent of the retainer design [3, 44]. Collectively, these findings suggest that both CAD/CAM‑fabricated and robotically bent retainers provide reliable mid‑term stability with minimal clinically relevant differences. Nonetheless, the slightly enhanced dimensional accuracy observed in digitally or robotically manufactured retainers supports robotically bent designs as a cost‑efficient and clinically reliable alternative to fully CAD/CAM‑fabricated systems.

Despite these advantages, none of the retainer designs completely prevented three-dimensional tooth movements. This supports the concept that fixed retainers cannot be regarded as fully passive over time and may become biologically or mechanically activated due to functional loading, material properties, or degradation at the adhesive interface [45–47].

In this context, bonding failures played a critical role: in the present cohort, bonding site defects were significantly associated with severe tooth position changes and occurred on average approximately one year after retainer placement. Bonding failures were observed more frequently in CAD/CAM‑fabricated and robotically bent retainers than in conventionally fabricated multistranded Twistflex retainers. Notably, a considerable proportion of these failures occurred within the first 6 months after placement, which may indicate a phase of increased susceptibility related to early clinical or patient-related factors. Importantly, recent clinical reports indicate that even subclinical micro‑defects at the composite–wire interface can generate unintended force systems capable of inducing clinically significant tooth movements, including X‑effects and wire‑syndrome‑like patterns, underlining the need for careful, long‑term monitoring of bonded retainers [48]. The low prevalence of clinically visible wire syndrome observed in the present study is therefore likely related to the relatively short to mid-term observation period. Previous reports indicate that such pronounced iatrogenic movements are rare but typically emerge after longer retention intervals, often several years after retainer placement [5, 10, 49]. Consequently, the absence of overt X-effects in the current cohort should not be interpreted as evidence of long-term retainer passivity but rather underscores the need for extended longitudinal follow-up.

### Limitations

There are a few limitations to note. Its retrospective design reduces control over potential confounders. Group allocation was based on patients’ individual preference for or against fixed retention, which represents a substantial source of selection bias: patients declining fixed retainers may differ systematically from those accepting them with respect to compliance behavior, oral hygiene attitudes, or socioeconomic factors; characteristics that are likely to influence removable retainer wear adherence as well. This potential confounding by indication limits causal interpretation of the observed stability differences between the two groups. Furthermore, compliance with removable retainers could not be objectively verified, which represents an inherent limitation of all removable retention systems, as wear adherence is ultimately dependent on patient behavior and cannot be controlled irrespective of study design. Importantly, this compliance dependency is not a methodological shortcoming of the present study but reflects a characteristic of removable appliances. Therefore, the independence from patient cooperation represents a fundamental advantage of fixed retention over removable appliances. In addition, because group 1 includes multiple fixed retainer designs under a single exposure category, the primary fixed-versus-removable comparison reflects a mixed protocol; readers should interpret it accordingly.

Although three‑dimensional superimposition enabled detailed quantification of tooth movements, distinguishing relapse from retainer‑associated effects was not always possible. Vertical changes should also be interpreted with caution, as superimposition on molars and premolars may cause physiological settling to appear as relative anterior displacement. Furthermore, different retainer types were included and analysed, however, subgroup comparisons were limited by sample size. Larger studies are needed to enable meaningful comparisons. Finally, the follow‑up period reflects short‑ to mid‑term retention, and longer observation is required to assess long‑term stability.

## Conclusions

Clinically relevant tooth position changes may occur during orthodontic retention and are not confined to the early post-treatment phase. In particular, vertical control of the anterior teeth remains a critical challenge. Based on the results it can be summarized:

- When comparing retention protocols, patients with combined fixed and removable retention (group 1) demonstrated greater overall stability than patients with only removable retention (group 2), particularly in the mandibular arch. Based on these findings, the use of fixed retention in addition to removable appliances is recommended.
- Nevertheless, vertical positional instability of anterior teeth may occur during retention, irrespective of the retention protocol.
- In a subgroup analysis of fixed retainer designs all evaluated designs were broadly comparable in maintaining stability. Digitally fabricated and robotically bent retainers showed statistically significantly better control of incisor angulation and tended toward lower dimensional variability, though differences were overall modest. However, severe tooth position changes might occur predominantly in the presence of bonding defects.
- Prolonged and regular monitoring of the retention phase is essential, as tooth position changes and retainer-related complications may develop over time.

## Supplementary Information

Below is the link to the electronic supplementary material.

Supplementary Material 1

## Data Availability

No datasets were generated or analysed during the current study.
